# Severe Thyrotoxicosis Caused by Molar Pregnancy: A Case Report and Review of the Literature

**DOI:** 10.7759/cureus.37582

**Published:** 2023-04-14

**Authors:** Christina Frasik, Eli Luong, Melissa Chang, Sareen Sandhu, Aalap C Shah

**Affiliations:** 1 Obstetrics and Gynecology, University of California, Irvine School of Medicine, Irvine, USA; 2 Anesthesiology and Perioperative Medicine, University of California, Irvine School of Medicine, Irvine, USA; 3 Endocrinology, University of California, Irvine School of Medicine, Irvine, USA

**Keywords:** multidisciplinary care team, hyperthyroidism, hypermetabolic state, molar pregnancy, thyrotoxicosis

## Abstract

Severe thyrotoxicosis is an acute and life-threatening state of hyperthyroidism. While it is a rare presentation of hyperthyroidism, it is clinically significant because of its high mortality and necessitates early identification and treatment to reduce the incidence of poor outcomes. The most common causes of this hypermetabolic state are Graves’ disease, toxic thyroid adenoma or multinodular goiter, thyroiditis, iodine-induced hyperthyroidism, and excessive intake of levothyroxine. The less common causes include trauma, medications (i.e., amiodarone), discontinuation of anti-thyroid medications, and interactions with sympathomimetic medications such as ketamine that may be administered during general anesthesia. Regardless of etiology, thyrotoxicosis management should be coordinated using an interdisciplinary team-based approach to optimize outcomes.

We discuss a molar pregnancy requiring emergency surgery as an uncommon cause of thyrotoxicosis and highlight appropriate management steps. The patient’s symptoms resolved post-operatively, and her post-operative laboratory results (thyroid function and beta-human chorionic gonadotropin {β-hCG}) were followed until they normalized. The patient’s preoperative presentation and preparation with a multidisciplinary team discussion, intraoperative anesthetic considerations and course, and post-operative management and follow-up are described.

## Introduction

Hydatidiform moles are a subset of gestational trophoblastic disease, formed through errors in gametogenesis or fertilization. Complete molar pregnancies are the result of the overexpression of paternal genes, with resultant 46,XX or 46,XY pregnancies. These genetic aberrations with resultant increased beta-human chorionic gonadotropin (β-hCG) lead to their characteristic appearance, including hydropic villi with absent fetal tissue, and symptomatology is encountered clinically [1]. In uncomplicated cases of complete molar pregnancies, patients frequently present with vaginal bleeding, abdominal pain or cramping, excessive vomiting, and a uterus measuring larger than gestational age. While less commonly seen, some patients present with symptoms suggestive of increased thyroid hormone levels secondary to increased β-hCG levels, in extreme cases causing thyrotoxicosis or thyroid storm, with symptoms such as significant tachycardia, fever, agitation, and altered mental status. Given the differential diagnoses and variable time course of their related signs and symptoms, patients may present and expeditiously transition through different phases of the healthcare system, from ambulatory, urgent care, or emergency-level setting to inpatient admission, the operating room (OR), and even the intensive care unit (ICU). We describe a case in which a patient presents in the outpatient setting and rapidly deteriorates, requiring expeditious transfer of care and the assembly of a multidisciplinary team equipped to bring a patient with thyrotoxicosis to the operating room for the management of a complete molar pregnancy. The CAse REport (CARE) guidelines were referenced in creating this case report [1]. A written Health Insurance Portability and Accountability Act authorization was obtained from the patient.

## Case presentation

A 36-year-old G3P2002 female presented to the outpatient setting for a second evaluation of a newly diagnosed complete molar pregnancy following an ultrasound demonstrating an enlarged uterus for gestational age, a “snowstorm” appearance on imaging, the absence of embryonic tissue, and ovarian theca-lutein cysts bilaterally. Of note, the patient required a second evaluation following refusal of care and subsequent transfer from a local Catholic hospital for the management of molar pregnancy. She reported a subjective fever, significant nausea, and vomiting and was tachycardic, prompting a transition to the emergency department (ED) for higher-level care. Once in the ED, the patient was found to be tachycardic with a peak heart rate (HR) of 140, along with a temperature of 39.6°C and a normal chest radiograph. β-hCG was found to be >270,000 mIU/mL, outside of our laboratory’s range of discrete measurement, and thyroid function tests were significant for thyroid-stimulating hormone (TSH) of <0.010 µIU/mL and free thyroxine (T4) of 3.45 ng/dL. A calculated Burch-Wartofsky score of 35 was concerning for impending thyroid storm secondary to elevated β-hCG.

Given the concern for molar pregnancy, the patient was emergently taken to the OR for the dilation and evacuation of molar pregnancy prior to initiating therapy for a suspected thyroid storm. Notable vital signs prior to anesthetic induction included sinus tachycardia with a heart rate (HR) of 133 beats per minute (bpm), blood pressure (BP) of 131/72 mmHg, and temperature of 37.4°C. An invasive arterial catheter was placed for close hemodynamic monitoring and an additional intravenous catheter for continuous infusions. After preoxygenation, the patient underwent an uneventful general anesthetic induction with propofol, fentanyl, and rocuronium, followed by atraumatic direct laryngoscopy and intubation. The maintenance of anesthesia was achieved with inhaled sevoflurane titrated to one minimum alveolar concentration (MAC).

Intravenous hydrocortisone was administered to decrease the peripheral conversion of free T4 to triiodothyronine (T3) upon arrival at the operating room. Dexamethasone was subsequently administered after the evacuation of the molar pregnancy to continue to decrease peripheral free T4 conversion, although it was not continued during the post-operative phase or in telemetry. Prophylactic azithromycin was administered over 30 minutes prior to the start of the procedure. An esmolol infusion was initiated at 50 mcg/kg/minute and titrated to as high as 200 mcg/kg/minute to treat tachycardia prior to and during the operation, which commenced after sustaining an HR of <100 bpm. A brief period of hypotension occurred immediately after the evacuation of the molar pregnancy, with a BP nadir of 75/38 mmHg, prompting the cessation of the esmolol infusion and phenylephrine infusion and the titration of inhaled sevoflurane. The rate was decreased and then stopped within 10 minutes of the evacuation of the hydatidiform mole and was not restarted afterward or during the post-operative period. Oxytocin was administered to facilitate uterine contraction after evacuation. The total estimated blood loss was 100 mL. At the conclusion of the surgery, inhaled sevoflurane anesthesia was discontinued, and the patient emerged from anesthesia with a smooth extubation to room air. The last recorded intraoperative esophageal temperature prior to extubation was 36.8°C. There was no recurrence of tachycardia as the patient was transported to the post-anesthesia care unit (PACU). After 45 minutes of observation in the PACU (1:2 nurse/patient ratio), the patient was transferred to a telemetry unit for close observation and monitoring for 24 hours prior to transfer to the post-operative inpatient ward.

Propylthiouracil (PTU) was started at 200 mg every four hours with the first dose administered intraoperatively. In the days following the evacuation, thyroid function tests improved with an associated decline in β-hCG levels. On the day of discharge (post-operative day 4 {POD4}), free T4 declined to 3.87 ng/dL (from a peak of 4.17 ng/dL), and T3 was 3.1 pg/mL. Clinically, the patient was asymptomatic, and PTU was discontinued prior to discharge from the hospital. Oral propranolol was administered every four hours starting on post-operative day 1 (POD1) and discontinued on an as-needed basis for palpitations. She was seen in the endocrinology clinic two weeks post discharge and was found to have a normal of thyroid function tests (Table 1).

**Table 1:** β-hCG, free T4, TSH, and free T3 over time β-hCG, beta-human chorionic gonadotropin; TSH, thyroid-stimulating hormone; POD, post-operative day; T4, thyroxine; T3, triiodothyronine

POD	β-hCG (mIU/mL)	Free T4 (ng/dL)	TSH (µIU/mL)	Free T3 (pg/mL)
0	>270,000	3.62	<0.009	
1	>270,000	3.92	<0.008	
2	134,769	4.17	<0.007	
3	67,262	3.87	<0.006	3.1
4	34,878	2.74	<0.005	3.2
5	24,183	2.39	<0.004	3.4
13	868			
14	824	0.9	0.33	2
19	235	0.9	0.84	
26	72			
35	24			
42	12			
48	9			
64	4			
94	<3			

**Figure 1:**
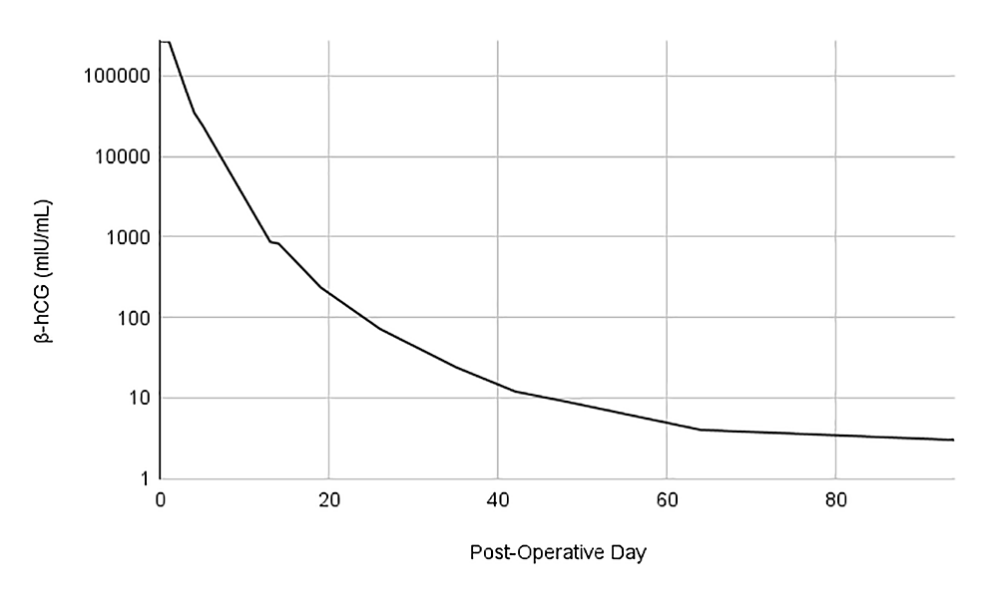
β-hCG normalization after the evacuation of hydatidiform mole β-hCG: beta-human chorionic gonadotropin

In addition, the gynecology team followed the patient’s β-hCG weekly after evacuation (Figure 1). After three months, the patient’s last two weekly β-hCG measurements were <5 mIU/mL, the laboratory result threshold for nonpregnant individuals. Ideally, laboratory results would have been followed for one additional week with β-hCG of <5 mIU/mL and followed monthly for six additional months. The patient, however, was lost to follow-up and ceased presenting for laboratory work.

## Discussion

Due to the similarity of the α subunit of β-hCG to TSH, clinical hyperthyroidism in pregnant patients is not an uncommon occurrence, with an incidence rate of approximately one in 100 [2,3]. The unique characteristics of β-hCG, specifically in trophoblastic disease, contribute to the extent of thyrotoxicosis that can be seen in these patients. The β-hCG molecules associated with trophoblastic disease are more heterogenous with degraded variations, which have been found to have 1.5-2 times more thyrotropic activity compared to physiologic β-hCG from pregnancy [4-6]. The presence of hydatidiform mole may also result in supranormal levels of β-hCG and can manifest as symptoms of severe hyperthyroidism. There is little consensus in the literature in regard to the relationship between the extent of HCG elevation and the severity of thyrotoxicosis. Cases have been reported of increased clinical severity and course complications with lower β-hCG levels when compared to their counterparts [7]. This favors physicians keeping the diagnosis of hyperthyroidism in mind without waiting for a specific β-hCG threshold to be reached.

Ideally, a hyperthyroid patient would first be treated to normalize thyroid function before surgery in cooperation with consultation from endocrinology. Since the removal of the hydatidiform mole would lead to the rapid resolution of symptoms, however, the anesthesiologist is faced with the management of a hyperthyroid patient at risk of transitioning to thyroid storm or thyrotoxicosis [8]. When conventional therapy fails, plasmapheresis has been successfully used in patients with severe hyperthyroidism due to hydatidiform mole for more rapid hormonal control [9]. Given the refusal of care at a prior hospital, the vital parameters, the significantly elevated β-hCG, and the availability of an operating room and emergency staff, an interdisciplinary decision was made to bring the patient to the OR for emergent evacuation. Additional staff members available to help with expedite care, including the preparation of vasoactive infusions and the placement of invasive catheters, allows for concurrent medical stabilization and avoids possible out-of-OR delays (i.e., pharmacy preparation and delivery).

The primary anesthetic management goals involve controlling the sympathetic stimulation secondary to hyperthyroidism. In these patients, the predominant physiologic derangement is the hypermetabolic state with resultant tachycardia caused by the direct action of circulating T3 on cardiac muscle. T3 also has a vasodilatory effect on the arteriolar smooth muscle cells [10]. In addition, other complications of a molar pregnancy include acute respiratory insufficiency, trophoblastic emboli and pulmonary edema, atrial fibrillation, heart failure, and disseminated intravascular coagulopathy [2]. Intraoperatively, a short-acting beta-blocker such as esmolol can be utilized as a continuous infusion or intermittent boluses to prevent tachycardia. Additionally, corticosteroids, such as hydrocortisone or dexamethasone, should be used to inhibit peripheral T4 to T3 conversion and can be rapidly administered as a part of the induction regimen by the anesthesia team during similar emergency cases [11]. Lastly, thioamides, such as methimazole or propylthiouracil, can be administered to decrease further production and the release of thyroid hormone, although the effect may only be beneficial after surgery since it takes 4-8 weeks to exert its effects on thyroid hormone production [2]. Invasive arterial line monitoring is mandatory in the presence of significant tachycardia and/or concern for intraoperative hypotension. Cardiovascular support would include volume expansion with crystalloids and phenylephrine as the vasopressor of choice to avoid further increasing tachycardia.

General anesthesia has been used successfully for the emergency management of a molar pregnancy in a number of case reports [12,13]. For anesthesia induction, non-depolarizing neuromuscular relaxants may be preferred over succinylcholine to avoid its histamine-releasing effects and associated tachycardia. Opioids to inhibit surgical stimulation are essential to avoid further sympathetic stimulation. For anesthesia maintenance, sevoflurane has been used in case reports, but its tocolytic effects may be undesirable [14]. The continuous infusion of propofol may be a more ideal choice, especially in combination with remifentanil to suppress sympathetic effects from circulating thyroid hormone. Aside from general anesthesia, regional anesthesia has been utilized successfully for the emergency management of a molar pregnancy. Spinal anesthesia is thought to be preferable than general anesthesia due to the benefits from sympathetic blockade and avoiding the tocolytic effect of volatile agents [15]. The benefits do need to be balanced against the complications of molar pregnancy as previously mentioned, in addition to patient anxiety during the procedure. Although symptoms may resolve soon after the removal of molar pregnancy, post-operative monitoring in a location with frequent hemodynamic monitoring such as the intensive care unit (ICU) or an observation telemetry unit, which was the disposition of our patient, is necessary as thyrotoxicosis may persist or worsen after surgery [12,14,16]. Thyroid hormones and β-hCG should be monitored for normalization and to exclude the presence of residual molar pregnancy tissue.

Molar pregnancy studies have found a logarithmic decline in β-hCG levels after evacuation [5,17,18]. The pattern typically follows an initial rapid reduction followed by a slower decline, as was seen in our patient. Schoeberl suggests that this can be explained by the dilution of β-hCG in two different tissue reservoirs (shallow and deep) [16]. During the slow decline phase, β-hCG levels can remain detectable for up to six months [19]. Previously, a six-month detectable β-hCG post evacuation was considered diagnostic for gestational trophoblastic neoplasm. The guidelines, however, were revised after studies showed that patients with a six-month detectable β-hCG had spontaneous normalization without treatment. These characteristics of HCG suggest that β-hCG levels (as well as thyroid function tests) will likely not be normalized at the time of discharge.

The management of thyrotoxicosis in trophoblastic disease poses a unique challenge given the time-sensitive nature of the situation as β-hCG levels are actively rising with the persistence of the molar pregnancy. Current guidelines typically recommend the normalization of thyroid function tests prior to surgery [20]. In the case of trophoblastic disease, however, the evacuation of the pregnancy is the definitive treatment posing a dilemma in regard to whether surgery should be delayed while thyroid hormone levels are attenuated. Case reports have been similar in the overall initial treatment strategy used for thyrotoxicosis in these situations (typically a combination of PTU/methimazole, steroids, and beta-blocker) [10,21]. However, variations arise in regard to the timing of surgery and whether to initiate long-term anti-thyroid medication after evacuation. There is limited literature offering guidance in these aspects of management. In our case, the decision was made not to pursue long-term anti-thyroid medication as the patient was asymptomatic and thyroid function levels were expected to normalize with associated decline in β-hCG. On follow-up, thyroid function tests were in fact within normal range suggesting that long-term continued treatment may not be necessary unless there is a rise in β-hCG levels.

## Conclusions

Diagnostic decision-making related to severe thyrotoxicosis in patients with hydatidiform moles can be confounded by the delayed presentation and rapid evolvement of nonspecific signs and symptoms related to a surge in β-hCG levels in these patients. Medical management is focused on decreasing the conversion and production of free thyroid hormone to halt the progression to an impending or actual thyroid storm. However, definitive treatment is focused on evacuating the molar pregnancy and removing the source of sympathomimetic hormones and the potential for continued hemodynamic instability.
